# Challenges in oral radiology teaching during COVID-19 pandemic

**DOI:** 10.1259/dmfr.20200178

**Published:** 2020-05-14

**Authors:** Maria Luiza Anjos Pontual, Eduarda Helena Leandro do Nascimento, Danyel Elias da Cruz Perez, Andrea Anjos Pontual, Flávia Moraes Ramos-Perez

**Affiliations:** 1 Department of Clinical and Preventive Dentistry, Universidade Federal de Pernambuco (UFPE), Recife, Pernambuco, Brazil

**Keywords:** COVID-19, Education, Oral Radiology, Teaching

COVID-19 is a highly transmissible infection caused by a novel RNA coronavirus (SARS-CoV-2). The pandemic COVID-19 infected more than 3.1 million people, with 220,000 deaths, as the April 29, 2020 (https://coronavirus.jhu.edu/map.html). To contain the spread of infection, isolating and social distancing measures have been determined by many countries.^1^ With the suspension of classroom lessons by most universities, the need to implement and upgrade distance learning has become essential and imperative. Thus, several challenges have arisen for maintaining teaching around the world during the pandemic of COVID-19.

Distance learning is defined as the education of students who may not be physically present. Currently, this process is made electronically, with the use of computers and networks.^2^ One of the concerns is that not all students have equity in internet access. Some students returned to their regions or even countries with restricted or poor internet access. The use of smartphones may facility this access mainly for those that do not have a personal computer. Additionally, teachers and students were taken by surprise and most of them need to adapt to the imposed reality. Indeed, the lack of prior training for effective web-based learning practices is worrisome.

There are a variety of programs and tools to help professors and students.^3^ In general, learning platforms offer the possibility to post the schedule of classes on the calendar, answer students' questions, and even discuss cases in chats functionality, besides the use of classes by video conferencing. In the absence of the availability of teaching platforms by the university, there is also the possibility of remote access classes by other programs such as Microsoft Teams^®^ (Microsoft Corp. Redmond, WA) and Google Hangout or Google Classroom, and the free, open source, end-to-end encrypted tool Jitsi^TM^ (Jitsi.org). To ensure privacy, the security of user data is essential.^4^ In this context, end-to-end encrypted open source tools like Jitsi are recommended. In end-to-end encrypted systems, only the users can read the messages. The data are encrypted in transit and at rest, and only the recipients are able to decrypt it. The information can be self-hosted, making it completely secure and independent on external manufacturers and servers. Thus, eventual concerns with the protection of patients' data (not just radiographic images, but clinical and sociodemographic information) could also be resolved, since the data protection rights from different countries are not allowing that patient data are transmitted to foreign servers which may be accessible by national governmental bodies or the like.

Oral Radiology teaching includes theoretical and practical classes with image interpretations and radiographic technique performing. The practical activities of interpretation can be done using social media and this one looks quite promising, especially at the time of COVID-19 pandemic. Instagram^®^, Facebook^®^, Youtube^®^, Twitter^®^, LinkedIn^®^^5^ and Pinterest^®^ are the platforms to be employed.^6^ A systematic review pointed out that E-learning (web-based learning or online learning) in Oral Radiology is as effective as traditional learning methods.^2^

Nevertheless, it is also important to use the platforms correctly and have enough time and expertise to implement them.^5^ The Pinterest^®^ serves only as a deep repository of radiologic images and the dissemination of case reports. On the other hand, other social media allow discussion and interaction between professors and students. This is interesting because the students who exercise become more familiar with the practice of “real-life” radiology.^7^ The Instagram stories option offers multiple-choice tools, polls, and more. Also, there is a possibility for the professor to make lives available and invite other speakers, making the subject more interesting and less formal. Consequently, social medias facility the feedback from the lecturer, and encourage active learning, all considered best practices for the teaching-learning process.^5^ Despite this, one of the biggest challenges is how to ensure the attention and effective involvement of the students, especially concerning practical activities of radiological interpretation. Also, the execution of radiographic techniques may become impractical.

As an alternative to perceiving and controlling students' learning about image interpretation, professors may require that they perform group or individual interpretation essay activities, preferably with time control to perform this task. In contrast, an even greater challenge will certainly be carrying out practical activities involving patient care and radiographic techniques. These activities should likely be replaced after the period of rigid social distancing. We also emphasize that the implementation of these post-pandemic activities should undergo changes related to biosafety procedures, which will certainly undergo some changes so that their care is maximized.

Therefore, teaching should be adapted to the new reality with the use of tools to make distancing learning more dynamic, interactive, and appropriate for the millennial generation.^3^ In addition, it is worth noting that new COVID-19 waves are not ruled out, with the possibility of further isolation and social distancing periods in the next 2 years.^1^ After the COVID-19 era, teaching in Oral Radiology will not be the same. Many changes will occur not only in issues related to cross-infection control but also in pedagogical issues with curricular changes.
